# Riboflavin Inhibits Histamine-Dependent Itch by Modulating Transient Receptor Potential Vanilloid 1 (TRPV1)

**DOI:** 10.3389/fnmol.2021.643483

**Published:** 2021-06-18

**Authors:** Kihwan Lee, Young In Choi, Sang-Taek Im, Sung-Min Hwang, Han-Kyu Lee, Jay-Zoon Im, Yong Ho Kim, Sung Jun Jung, Chul-Kyu Park

**Affiliations:** ^1^Tooth-Periodontium Complex Medical Research Center (MRC), Department of Physiology, School of Dentistry, Seoul National University, Seoul, South Korea; ^2^Department of Physiology, College of Medicine, Hanyang University, Seoul, South Korea; ^3^Gachon Pain Center and Department of Physiology, College of Medicine, Gachon University, Incheon, South Korea

**Keywords:** riboflavin, TRPV1, itch, histamine, channel blocker

## Abstract

Riboflavin, also known as vitamin B_2_, isfound in foods and is used as a dietary supplement. Its deficiency (also called ariboflavinosis) results in some skin lesions and inflammations, such as stomatitis, cheilosis, oily scaly skin rashes, and itchy, watery eyes. Various therapeutic effects of riboflavin, such as anticancer, antioxidant, anti-inflammatory, and anti-nociceptive effects, are well known. Although some studies have identified the clinical effect of riboflavin on skin problems, including itch and inflammation, its underlying mechanism of action remains unknown. In this study, we investigated the molecular mechanism of the effects of riboflavin on histamine-dependent itch based on behavioral tests and electrophysiological experiments. Riboflavin significantly reduced histamine-induced scratching behaviors in mice and histamine-induced discharges in single-nerve fiber recordings, while it did not alter motor function in the rotarod test. In cultured dorsal root ganglion (DRG) neurons, riboflavin showed a dose-dependent inhibitory effect on the histamine- and capsaicin-induced inward current. Further tests wereconducted to determine whether two endogenous metabolites of riboflavin, flavin mononucleotide (FMN) and flavin adenine dinucleotide (FAD), have similar effects to those of riboflavin. Here, FMN, but not FAD, significantly inhibited capsaicin-induced currents and itching responses caused by histamine. In addition, in transient receptor potential vanilloid 1 (TRPV1)-transfected HEK293 cells, both riboflavin and FMN blocked capsaicin-induced currents, whereas FAD did not. These results revealed that riboflavin inhibits histamine-dependent itch by modulating TRPV1 activity. This study will be helpful in understanding how riboflavin exerts antipruritic effects and suggests that it might be a useful drug for the treatment of histamine-dependent itch.

## Introduction

Riboflavin, also known as vitamin B_2_, consists of a group of water-soluble organic compounds that play a role in a wide range of cellular, tissue-growth, and developmental functions (Altun and Kurutas, 2016; Schwechheimer et al., 2016; Saedisomeolia and Ashoori, 2018). Moreover, it is found in food, including eggs, green vegetables, milk and other dairy products, meat, mushrooms, and almonds, and is used as a dietary supplement. Its deficiency (also called ariboflavinosis) results in some skin lesions and inflammations, such as stomatitis, cheilosis, oily scaly skin rashes, and itchy, watery eyes (Schmelz et al., 1997). In particular, riboflavin has been demonstrated to have anti-inflammatory, anti-nociceptive, anti-hyperalgesic, and anti-allodynic effects in inflammatory and neuropathic pain models (Franca et al., 2001; Granados-Soto et al., 2004; Bertollo et al., 2006), as well as antioxidant, anti-aging (Zou et al., 2017), and anticancer properties (Naseem et al., 2015). Recently, it was reported that riboflavin reduces hyperalgesia and inflammation in rats (Bertollo et al., 2006), which might involve the inhibition of synthesis and/or action of inflammatory mediators. However, in the formalin test, only the second phase of the formalin-induced pain response was inhibited (Franca et al., 2001; Granados-Soto et al., 2004). Although some of the analgesic effects of riboflavin on pain are known, its effect on itching due to skin problems is not yet known.

Itch (known as pruritus) and pain are the unpleasant sensations that frequently provoke scratching or are associated with actual or potential tissue damage, respectively. Both are also mediated by primary sensory neurons, such as the dorsal root ganglion (DRG) or the trigeminal ganglion (Ikoma et al., 2006). Itch is exclusively sensed as an irritation in the skin that causes a desire to scratch, whereas pain can be experienced almost all over the body (Yosipovitch et al., 2003). Itch is also a symptom of many common diseases, including diabetes mellitus, atopic dermatitis, and inflammatory, metabolic, neurologic, thyroid, and psychiatric diseases (Stander et al., 2003). People with chronic itch experience a decreased quality of life because of psychological disturbances often associated with the condition, including sleeplessness, anxiety, and depression (Dhand and Aminoff, 2014). Several substances are known to act as mediators and signaling pathway molecules during the pathogenesis of chronic itch (Song et al., 2018); however, the mechanisms involved remain unclear.

Histamine is one of the best-known mediators of cutaneous, pro-inflammatory conditions such as cutaneous mastocytosis, insect-bite reactions, urticaria, and drug rashes, all of which can cause itch (Moore et al., 2018). It is stored in basophilic leukocytes and mast cells and its release is triggered by a variety of itch stimuli, exciting a subset of unmyelinated C-fiber mechano-insensitive primary afferent neurons *via* the histamine-dependent (histaminergic) signaling pathway (Jian et al., 2016). Furthermore, the itch is often divided into histamine-dependent and histamine-independent subtypes (Ji et al., 2018). Histamine receptors are the members of G-protein-coupled receptors (GPCRs), and four subtypes (H1–H4) have been identified (Huang and Thurmond, 2008; Shim and Oh, 2008). More specifically, several studies have found that histamine H1 and H4 receptors can be detected on DRG neurons and are involved in histamine-induced itch (Han et al., 2006). Histamine in the skin is mainly supplied through the degranulation of mast cells. Tryptase secreted from the activated mast cells induces an itching sensation by activation of proteinase-activated receptor 2 (PAR2) in C-nerve fibers. Meanwhile, another intracellular mechanism is an increase in the excitability of cells through the activation of transient receptor potential vanilloid 1 (TRPV1) after the histamine that is released from mast cells binds to the histamine receptor present at the end of the C-nerve fiber, causing itching. Considering the results of the studies to date, the mechanism through which histamine-dependent itching activates TRPV1 has only been partially elucidated, suggesting that there is a possibility of developing antipruritic drugs targeting this marker.

TRPV1 is a nonselective cation channel that belongs to the family of transient receptor potential (TRP) channels and has been implicated in the mediation of pain and itch (Moore et al., 2018). This protein is modulated by GPCR signaling (Jian et al., 2016), and most itch stimuli activate GPCRs and trigger an itch sensation by activating TRP channels, including TRPV1 (Shim et al., 2007). Recent studies have demonstrated that the histamine H1 receptor (H1R) also activates TRPV1 *via* the production of 12-hydroxyeicosatetraenoic acid, a downstream metabolite of the PLA2 and lipoxygenase pathway in sensory neurons of the somatic nervous system (Shim et al., 2007). H1R is related to G_q_/G_11_ and activates phospholipase Cβ3 (PLCβ3), resulting in increased intracellular Ca^2+^ in DRG neurons *via* TRPV1 (Han et al., 2006). As with H1R, H4R also induces an increase in Ca^2+^ *via* the intracellular PLC signaling pathway and TRPV1, but not TRPA1 (Kim et al., 2004). In cultured sensory neurons, the highly selective TRPV1 antagonist capsazepine was found to significantly inhibit activation of the histamine H4R (Jian et al., 2016). In addition, TRPV1^−/−^ mice exhibit significantly attenuated scratching behavior after pro-itch stimulation (Costa et al., 2008). Therefore, TRPV1 is likely to play an important role in the induction of itch downstream of signaling through the activation of H1R or H4R and might thus be involved in the mechanism underlying the histamine-induced itch response. In the present study, regarding the effect of riboflavin on itch, we sought to assess its effects *in vivo* and *in vitro* based on behavioral and neurotransmission outcomes, respectively. We found that riboflavin inhibited histamine-induced currents *via* TRPV1 in small-sized DRG neurons and had an antipruritic effect in a mouse model of histamine-induced itch.

## Materials and Methods

### Animals

All experimental methods were approved by the Experimental Animal Ethics Committee of Hanyang University. Male C57BL/6 wild-type mice (OrientBio, Sungnam, Korea) weighing 18–22 g were used in the present study. All experimental animals were housed in a conventional facility with a 12:12-h light/dark cycle (lights on at 8 am) and *ad libitum* access to water and chow. Temperature and humidity were maintained at 22°C and 60%, respectively. All animals were allowed to habituate to the new environment for 1 week prior to any experiment.

### Drugs

Riboflavin, histamine, capsaicin, capsazepine, flavin mononucleotide (FMN), and flavin adenine dinucleotide (FAD) were purchased from Sigma-Aldrich. The stock solutions were prepared in ethanol or DMSO and stored at −20°C. The drugs were diluted to their final concentration with the extracellular solution for *in vitro* experiments or with saline for *in vivo* studies.

### Cell Culture and Transfection

HEK293 cells were maintained in DMEM with 10% fetal bovine serum at 37°C in a humidified atmosphere with 5% CO_2_. For transient transfection, cells were grown to approximately 70% confluence in 6-well plates. The next day, 1 μg/well of the *Trpv1* cDNA construct was transfected into cells with the Lipofectamine 2000 reagent (Invitrogen, Carlsbad, CA, USA) according to the manufacturer’s protocol. After 18–24 h, the cells were trypsinized and used for experiments.

### Primary DRG Neuron Culture

DRGs from all spinal levels of 6–8-week-old C57BL/6 mice (male) were removed aseptically and incubated with collagenase (5 mg/ml, Roche, Basel, Switzerland)/dispase-II (1 mg/ml, Roche, Basel, Switzerland) at 37°C for 40 min and then digested with 2.5% trypsin (Invitrogen, Carlsbad, CA, USA) for 7 min at 37°C, which was followed by the addition of 0.25% trypsin inhibitor (Sigma-Aldrich, St. Louis, MO, USA). Cells were mechanically dissociated using a flame-polished Pasteur pipette in the presence of 0.05% DNase I (Sigma-Aldrich, St. Louis, MO, USA). DRG cells were first plated on bare glass coverslips and then transferred to poly-D-lysine-coated coverslips. DRG cells were then incubated in a 5% CO_2_ atmosphere at 37°C and were maintained for 24 h before use.

### Itch Test

One day before the experiment, the animal’s nape hair was shaved to create a site of 1.5 cm in diameter for intradermal histamine injection. Experimental animals were placed in a small plastic cylinder (20 cm in diameter, 25 cm in height) with a pad to absorb any excrement for 15 min to allow them to acclimate, and then, histamine (100 μg/50 μl, Sigma-Aldrich, St. Louis, MO, USA) was injected intradermally into the nape of the animal’s neck. To measure the number of rear hind leg direct scratching events, experimental animals were monitored for 15 min before intradermal injection and for 30 min after injection. A direct scratching event involved the animal lifting its hind paw to the injection site, scratching it, and then returning the paw to its original position. To assess particular receptor responses to riboflavin, the same procedure, described herein, was followed, and behavioral responses were observed.

### Rotarod Locomotion Test

To determine whether riboflavin affects animal locomotor activity, mice were placed on a horizontal bar rotating at a constant speed of 4 RPM using a rotarod apparatus (ROTA-ROD, B.S Techno lab INC., Seoul, Korea). Twenty-four hours before rotarod testing, all mice were preliminarily tested, and only those that were able to remain on the rod for at least 120 s were included in the study. Time spent on the bar (in seconds) and the number of falls were measured over a 2 min trial. Scores were then compared and analyzed based on animal performance 5 min before and 30 min after treatment with vehicle or riboflavin. The test was repeated three times and the mean value for each animal was calculated.

### *Ex vivo* Recordings

Mice were euthanized by CO_2_ inhalation, and the hairy skin of the hind paw innervated by the saphenous nerve was dissected from attached connective tissues, muscles, and/or tendons. We used an organ bath that consisted of two chambers (a perfusion chamber and a recording chamber) separated by an acrylic wall. The perfusion chamber was continuously perfused with synthetic interstitial fluid (SIF; in mM: 3.5 KCl, 107.8 NaCl, 0.69 MgSO_4_·7H_2_O, 1.53 CaCl_2_·2H_2_O, 1.67 NaH_2_PO_4_·2H_2_O, 26.2 NaHCO_3_, 9.64 C_6_H_11_NaO_7_, 7.6 sucrose, and 5.55 glucose) saturated with a mixture of 95% O_2_ and 5% CO_2_ and maintained at 31 ± 1°C. After dissection, the preparation was placed with the epidermal side down, and nerves attached to the skin were drawn into the recording chamber, which was filled with paraffin oil. The nerve was placed on a fixed mirror, its sheath was removed, and nerve filaments were repeatedly teased apart such that single fiber recordings could be obtained using two gold electrodes, one for recording and the other for reference. Spikes from single C-fibers were recorded extracellularly with a differential amplifier (DP311; Warner Instruments, Hamden, CT, USA), transferred to a computer *via* a data acquisition system (DAP5200a; Microstar Laboratories, Inc., Bellevue, WA, USA), and analyzed offline using a window discrimination software package (Dapsys 8; Bethel University, Arden Hills, MN, USA^1^).

### Electrophysiology

Whole-cell current-clamp recordings were performed at room temperature using the HEKA EPC10 amplifier (HEKA Elektronik GmbH, Lambrecht/Pfalz, Germany). Patch pipettes were pulled from borosilicate capillaries (Chase Scientific Glass Inc., Rockwood, CA, USA). When filled with the pipette solution, the resistance of the pipettes was 4–6 MΩ. The recording chamber was continuously perfused (2 ml/min). We compensated for series resistance (>80%), and leak subtraction was performed. The Pulse v8.30 software (HEKA) was used during experiments and for analyses. The internal pipette solution was composed of the following (in mM): 140 KCl, 1 CaCl_2_, 2 MgCl_2_, 10 EGTA, 10 D-glucose, and 10 HEPES adjusted to a pH of 7.3 with NaOH, with an osmolarity of 295–300 mOsm. The extracellular solution contained the following (in mM): 140 NaCl, 5 KCl, 2 CaCl_2_, 1 MgCl_2_, 10 HEPES, and 10 D-glucose, adjusted to a pH 7.3 of with NaOH, with an osmolarity of 300–310 mOsm. All drugs used in this experiment were dissolved in an extracellular solution. Voltage-clamp experiments were performed at a holding potential of −60 mV.

### Data Analyses

All data were analyzed using the SPSS 24 software (IBM, Armonk, NY, USA). Differences between the two datasets were evaluated by paired or unpaired *t*-tests. Differences between multiple groups were evaluated by one-way ANOVA followed by Tukey’s *post hoc* tests. Differences were considered significant when the *p-value* was less than 0.05 (*p* < 0.05). All data are expressed as means ± standard errors of the mean (SEMs). Dose-response analyses were performed using the Origin 6.1 software (MicroCal, Northampton, MA, USA).

## Results

### Effect of Riboflavin on Behavioral Motor Function and Scratching

**Figure 1:**
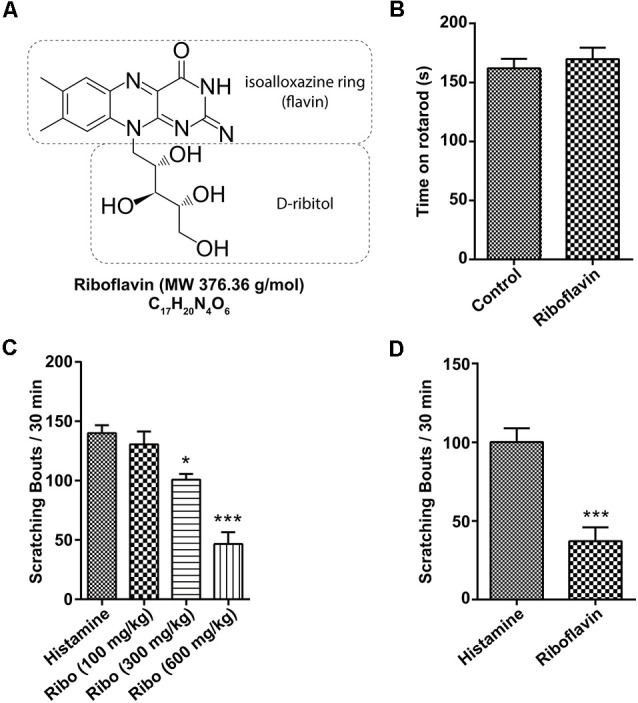
Effect of riboflavin on itching caused by histamine. **(A)** Molecular structure of riboflavin. **(B)** Motor function assessed by fall latency on the rotarod test after the oral administration of vehicle or riboflavin (600 mg/kg, *n* = 3). Total scratching bouts during the first 30 min after **(C)** the oral administration of riboflavin (each 100, 300, and 600 mg/kg, *n* = 5) or **(D)** the intradermal injection of riboflavin (1 μM, *n* = 5, *p < 0.05, ***p < 0.001). Ribo, riboflavin.

Riboflavin (known as vitamin B2) is a water-soluble member of the B-vitamin family found in food and used as a dietary supplement with a molecular weight (MW) of 376.4 g/mol (Figure 1A). In previous studies, riboflavin was found to be associated with motor function and anti-allergic effects in several diseases (Morawiecki, 1955; Coimbra and Junqueira, 2003; Bashford et al., 2017; Naghashpour et al., 2019). Because high doses of riboflavin promote the recovery of some motor functions in Parkinson’s disease (Coimbra and Junqueira, 2003), we first performed the rotarod test to exclude the influence of motor activity on behavioral assessments in the present study. As shown in Figure 1B, both animals orally administered riboflavin (169.7 ± 9.8 s, *n* = 3) and controls (162.0 ± 8.0 s, *n* = 2) had similar fall latencies in the rotarod test. Meanwhile, to measure the anti-allergy effect, we evaluated the number of scratching events induced by the intradermal injection of histamine (100 μg/50 μl) as one sign of an allergic reaction (Morawiecki, 1955), which is 140.0 ± 6.6 bouts / 30 min (*n* = 5). The oral administration of high-dose riboflavin (600 mg/kg) significantly reduced the total number of scratching (46.6 ± 9.9 bouts / 30 min, *n* = 5) over the observation period in a dose-dependent manner (130.6 ± 10.8 and 100.8 ± 4.8 bouts / 30 min in 100 mg/kg and 300 mg/kg, respectively, *n* = 5; Figure 1C). In addition, intradermal injections of riboflavin also significantly reduced scratching behavior (37.6 ± 8.8 bouts / 30 min, *n* = 5) compared to histamine (100.6 ± 8.7 bouts / 30 min, *n* = 5; Figure 1D).

### Effect of Riboflavin on Histamine-Induced Peripheral Neuron Discharge

**Figure 2:**
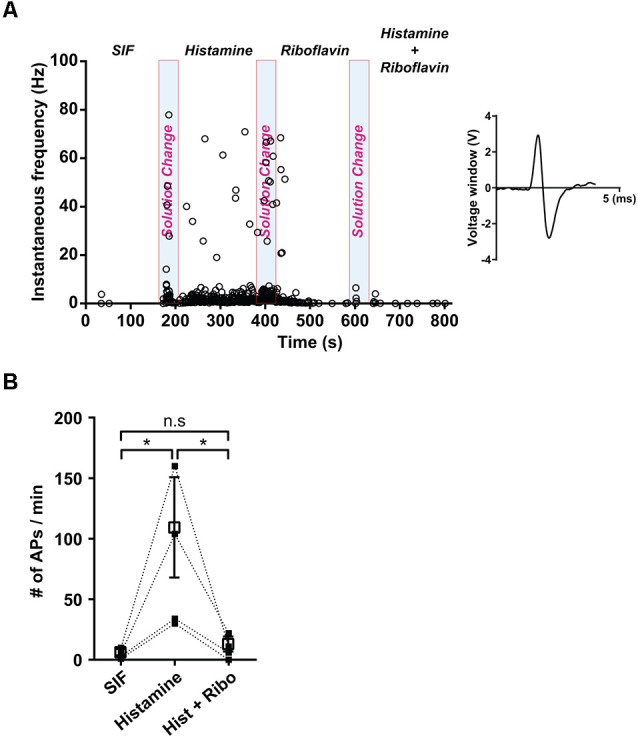
Effect of riboflavin in histamine-induced peripheral sensory neural discharge. **(A)** The instantaneous response frequency from a representative single C-fiber with the application of synthetic interstitial fluid (SIF), histamine (500 μM), and a combination of histamine (500 μM) and riboflavin (100 μM). Based on the AP’s shape, the number of generated nerve discharges was measured and the shape of AP was shown in the inset panel. Light blue shaded bars represent each period of solution change with the application of SIF, histamine, and a combination of histamine and riboflavin. **(B)** Spike counts from a single fiber during the control period of SIF administration and a corresponding histamine and riboflavin responsive period (*p < 0.05, *n* = 4). Open square represents mean of number of APs with SIF, histamine, and a combination of histamine and riboflavin. AP, action potential; Hist, histamine; Ribo, riboflavin; n.s., not significant.

Next, we prepared *ex vivo* recordings to examine the propagation of extracellular action potential (AP) along a single nerve fiber. Single fibers were stimulated with SIF, histamine, and riboflavin in succession. The application of histamine evoked the generation of AP, resulting in an increase to 82 ± 31.1 firing rates/min, *n* = 4, compared with the firing rates/min in SIF (4.8 ± 1.9 firing rates/min, *n* = 4; Figure 2A). Furthermore, when histamine was applied in combination with riboflavin, the propagation of APs was also inhibited. *Ex vivo* spike count analyses of single-fiber recordings revealed that riboflavin had a significant inhibitory effect on histamine-induced APs (9.8 ± 4.7 firing rates/min; Figure 2B).

### Inhibitory Effects of Riboflavin on Histamine- or Capsaicin-Induced Currents in Small-Sized DRG Neurons

**Figure 3:**
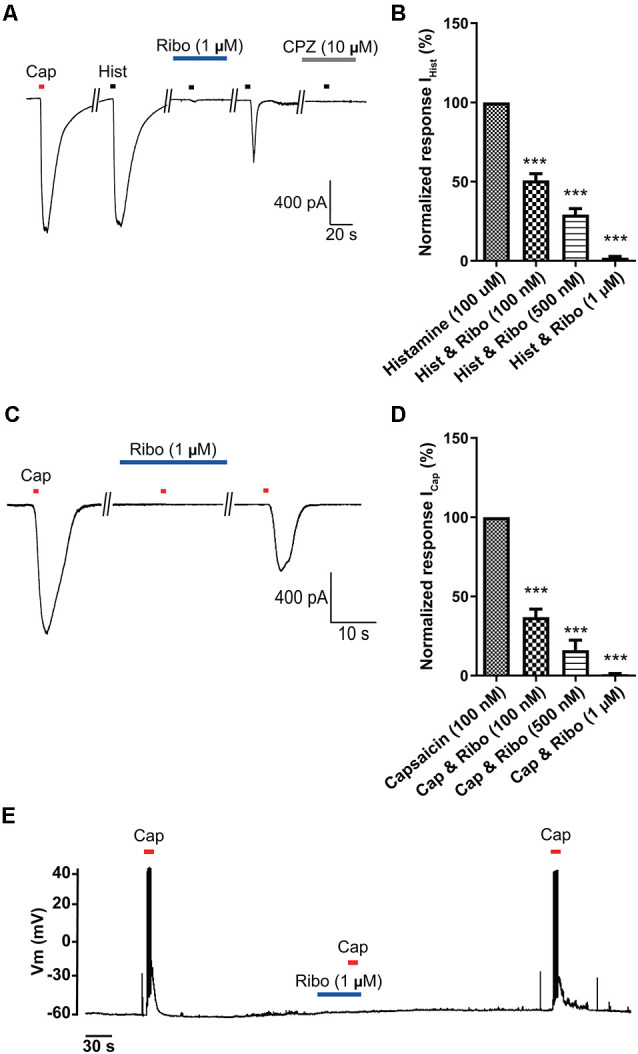
The inhibitory effects of riboflavin on histamine- or capsaicin-induced currents in small-sized dorsal root ganglion (DRG) neurons. **(A)** Inward currents were activated by capsaicin (Cap, 100 nM, 5 s or histamine (Hist, 100 μM, 5 s) that was blocked by riboflavin (1 μM, 5 min) or capsazepine (CPZ, 10 μM, 5 min) at the holding potential of −60 mV in small-sized DRG neurons. **(B)** Summary of I_Hist_ inhibition by riboflavin relative to the other I_Hist_ (***p < 0.001, *n* = 5, LSD’s *post hoc* after one-way ANOVA). **(C)** Capsaicin-induced inward currents were blocked by riboflavin (1 μM) at the holding potential of −60 mV in small-sized DRG neurons. **(D)** Summary of I_Cap_ inhibition by riboflavin relative to the others I_Cap_. Results are presented as the mean ± SEM (***p < 0.001, *n* = 5, LSD’s *post hoc* after one-way ANOVA). **(E)** Current-clamp recording showing the blockade of capsaicin (Cap, 200 nM)-induced action potentials by riboflavin (1 μM). Cap, capsaicin; Hist, histamine; Ribo, riboflavin; CPZ, capsazepine.

Even though it is well established that riboflavin elicits a protective effect on DRG neurons (Salman et al., 2015), its effects on itch in small-sized DRG neurons as well as its mechanism are still not known. Therefore, we evaluated the potential effects of riboflavin on histamine- and capsaicin- evoked inward currents in small-sized DRG neurons using a whole-cell patch-clamp recording technique. As shown in Figure 3A, current responses to capsaicin and histamine were similar in amplitude under the voltage-clamped condition at −60 mV, and pretreatment with riboflavin (1 μM) significantly reduced histamine-induced currents (1.9 ± 0.9%, *n* = 5) in a dose-dependent manner (50.7 ± 4.2% and 29.1 ± 3.8% in 100 nM and 500 nM riboflavin, respectively, *n* = 5, Figure 3B). Furthermore, after the removal of riboflavin, histamine evoked inward currents, despite a lack of recovery to normal control levels, which were completely inhibited by capsazepine, a TRPV1 antagonist (Figures 3A,B). This result was consistent with the previous study showing that histamine could activate TRPV1 (Shim et al., 2007). To further examine the effect of riboflavin on capsaicin-induced currents, it was applied prior to a second stimulation with capsaicin. In the DRG neuron shown in Figure 3C, capsaicin-induced inward currents were completely inhibited by riboflavin (0.9 ± 0.43% in 1 μM, *n* = 5) administration in a dose-dependent manner (36.9 ± 5.0% and 16.0 ± 6.4% in 100 nM and 500 nM, respectively, *n* = 5, Figure 3D). After a washout period, capsaicin-induced currents in these neurons were partially recovered. In addition, current-clamp recordings revealed that riboflavin (1 μM) also completely blocked capsaicin-evoked APs in small-sized DRG neurons (Figure 3E). These data indicate that riboflavin inhibits histamine- and capsaicin-induced inward currents in small-sized DRG neurons.

### Effect of Flavin Mononucleotide (FMN) and Flavin Adenine Dinucleotide (FAD) on Capsaicin-Induced Currents in Small-Sized DRG Neurons

**Figure 4:**
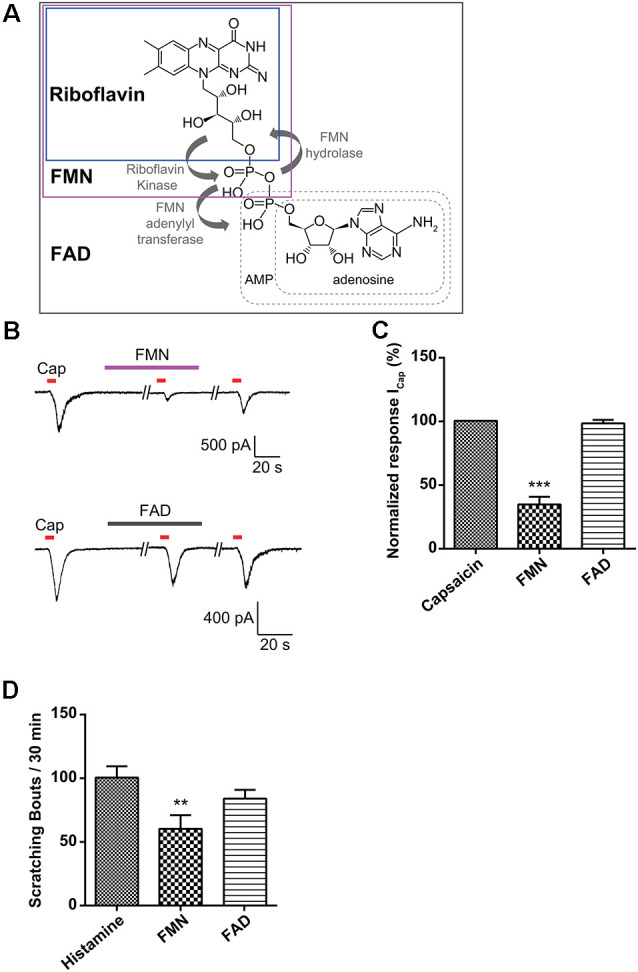
Effect of flavin mononucleotide (FMN) and flavin adenine dinucleotide (FAD) on capsaicin-induced currents in small-sized dorsal root ganglion (DRG) neurons. **(A)** Biosynthesis of FMN and FAD from riboflavin. **(B)** A representative trace of capsaicin (Cap, 200 nM)-induced inward current in the absence and presence of FMN (1 μM) or FAD (1 μM) are shown. **(C)** The relative peak amplitudes of capsaicin-induced inward currents after a second stimulation compared to those after the first stimulation of each neuron are shown (holding voltage at −60 mV), normalized to the current in the same cell (***p < 0.001, *n* = 8, LSD’s *post hoc* after one-way ANOVA). **(D)** The total scratching bouts during the first 30 min after the intradermal injection of FMN (1 μM) and FAD (1 μM) compared to those with histamine (100 μg/50 μl). Results are presented as the mean ± SEM (**p < 0.01, *n* = 4, LSD’s *post hoc* after one-way ANOVA). Cap, capsaicin; FMN, flavin mononucleotide; FAD, flavin adenine dinucleotide.

Riboflavin is composed of a dimethyl isoalloxazine nucleus (isoalloxazine is the basic structure of all flavin molecules) and ribitol (the reduced form of the sugar ribose; Figure 1A). Riboflavin is converted to FMN by the addition of a phosphate group on the ribityl side chain and is further converted to FAD by the addition of adenosine monophosphate (AMP; Figure 4A). In general, both FMN and FAD play a key role as cofactors for enzymes that catalyze certain complexes in the Krebs cycle that are involved in energy metabolism and are required for functions in numerous reduction and oxidation (redox) reactions in humans (Thakur et al., 2017). Therefore, along with the effect of riboflavin, we tested whether FMN and FAD could inhibit capsaicin-induced inward currents in small-sized DRG neurons. For this, FMN (1 μM) or FAD (1 μM) was applied 2 min prior to a second stimulation with capsaicin and continuously until 2 min after the removal of capsaicin. Interestingly, the capsaicin-induced inward current was markedly blocked by FMN (34.4 ± 6.0%, *n* = 8), but not by FAD (98.1 ± 2.7%, *n* = 8; Figures 4B,C). Furthermore, the intradermal injection of FMN (60.3 ± 10.8 bouts / 30 min, *n* = 4), but not FAD (84.0 ± 6.9 bouts / 30 min, *n* = 4), partly reduced the number of scratching responses compared to that with only histamine (100.6 ± 8.8 bouts / 30 min, *n* = 5; Figure 4D). This result suggests that a riboflavin metabolite, FMN, as well as riboflavin has an antipruritic effect.

### Effect of Riboflavin, FMN, and FAD on Capsaicin-Induced Currents in TRPV1-Transfected HEK293 Cells

**Figure 5:**
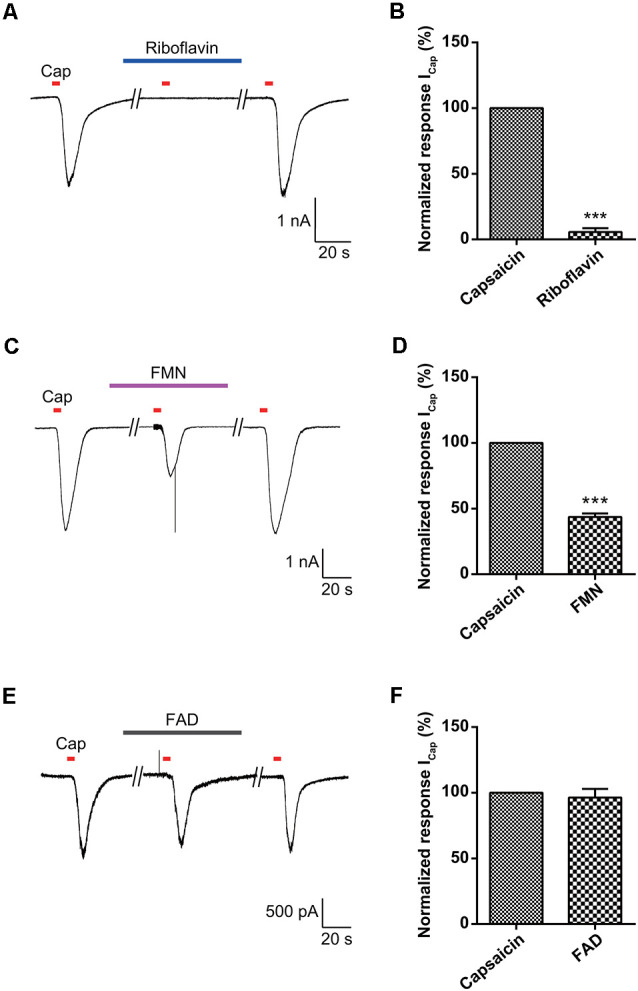
Effect of riboflavin, FMN, and FAD on capsaicin-induced currents in transient receptor potential vanilloid 1 (TRPV1)-transfected HEK293 cells. The responses to capsaicin (Cap, 200 nM) in the absence and presence of **(A)** riboflavin (1 μM), **(C)** FMN (1 μM), and **(E)** FAD (1 μM) are shown in TRPV1-transfected HEK293 cells. The normalized response of capsaicin currents induced by the second stimulation compared to those with the first stimulation in each neuron is shown **(B,D,F)**. Results are presented as the mean ± SEM (***p < 0.001, *n* = 3, unpaired *t*-test). Cap, capsaicin; FMN, flavin mononucleotide; FAD, flavin adenine dinucleotide.

To further assess the direct effects of riboflavin or its metabolites on activation of TRPV1, we examined the effects of riboflavin, FMN, and FAD on capsaicin-induced inward currents in TRPV1-transfected HEK293 cells. Riboflavin (1 μM), FMN (1 μM) or FAD (1 μM) were applied 2 min prior to a second stimulation with capsaicin and then continuously applied until 2 min after its removal. As shown in Figures 5A–D, the inward current induced by capsaicin in TRPV1-transfected HEK293 cells was completely or partially blocked by riboflavin (5.6 ± 2.9%, *n* = 3) and FMN (43.6 ± 2.6%, *n* = 3), respectively, while the capsaicin-induced current was not altered by FAD (96.3 ± 6.6%, *n* = 3; Figures 5E,F). In conclusion, these results indicate that riboflavin and FMN inhibit capsaicin-induced inward currents *via* a direct blocking activation of TRPV1 ion channel.

## Discussion

Itch is mainly mediated by unmyelinated, slow conducting C-fibers, which are found at the dermal–epidermal junction, with free nerve endings that extend into the epidermis (Schmelz et al., 1997; Mcneil and Dong, 2012; Cho et al., 2016). GPCRs or ion channels can initiate itch signals (Kremer et al., 2014) *via* proteases such as tryptase and neuropeptides, such as substance P, prostaglandins, and histamines (Mollanazar et al., 2016). Under pathological conditions such as nerve injury and tissue damage, the sensation of itch develops into a chronic condition resulting from excess peripheral firing or the attenuation of central inhibition of the itch-pathway neurons (Steinhoff et al., 2018). However, the diagnosis of chronic itch is challenging because different types (focal/widespread, peripheral/central) exist and there are few available treatment options (Lee et al., 2016). Currently, available strategies include treating or preventing the causal disease, such as diabetes or herpes zoster, and topical or systemic medication that attenuates excess neuronal firing (Luo et al., 2015). Unfortunately, the efficacy of these treatments is limited. Thus, it is necessary to develop more effective anti-pruritic medications for therapeutic applications to acute or chronic itch.

Histamine is known as the primary endogenous itch-inducing substance and is predominant in mast cells and basophile granulocytes (Mollanazar et al., 2016). Although antihistamines are often used for the treatment of itch (Yosipovitch and Bernhard, 2013), effective anti-pruritic therapeutics are still lacking for many types of itch (Song et al., 2018). In recent decades, great progress has been made toward revealing the molecular and cellular mechanisms for (acute) itch (Mishra and Hoon, 2013). Primary DRG sensory neurons are responsible for transmitting itch signals to the spinal cord dorsal horn and then projecting them to the brain (Han and Dong, 2014). Histamine binds H1 and/or H4 receptors to activate PLC3 and TRPV1 on free nerve terminals in the skin to elicit the itch sensation, which requires TRPV1-expressing C-fibers in the periphery (Imamachi et al., 2009). Therefore, an intracellular signaling pathway might converge on TRPV1 to mediate histamine itch response, although TRPA1-dependent and non-TRPV1–TRPA1-dependent itch pathways also exist (Luo et al., 2015).

TRPV1, a nonselective cation channel expressed predominantly in primary small-sized sensory neurons, is activated by capsaicin and acid or by temperatures >43°C (Caterina et al., 1997; Zygmunt et al., 1999). Further, TRPV1 sensitization induced by inflammatory mediators might contribute nociceptor hyperexcitability and neuropathic pain after spinal-cord injury (Wu et al., 2013). However, unlike the well-recognized role of TRPV1 in pain, its function in itch sensation has only recently been established (Luo et al., 2015). TRPV1-null mice show significantly attenuated itch behavior in response to the intradermal injection of histamine (Shim et al., 2007; Imamachi et al., 2009). In addition, histamine can activate TRPV1 channels expressed in HEK293 cells, and TRPV1 mediates the histamine-induced excitation of DRG neurons (Shim et al., 2007). The direct activation of TRPV1 induces pain, whereas the GPCR-mediated indirect activation of TRPV1 produces itch. Therefore, it appears that TRPV1 is differentially involved in pain and itch (Luo et al., 2015).

Vitamins are essential nutrients because they perform hundreds of roles in the body. Imbalances in vitamin absorption and deficiency can protect against or cause several diseases, respectively. In a previous study, some vitamins were used as diagnostic factors in chronic pruritus (Rajagopalan et al., 2017). One member of the vitamin B family, riboflavin, is also an essential component of normal cellular function and metabolism, acting to ameliorate neuroinflammation, glutamate excitotoxicity, mitochondrial dysfunction, and oxidative stress, which are involved in a wide array of diseases, such as migraine, anemia, cancer, diabetes mellitus, hypertension, Parkinson’s disease, and other neurological disorders (Mcnulty et al., 2006; Zhou et al., 2008; Long et al., 2012; Hwang, 2013; Dey and Bishayi, 2016; Marashly and Bohlega, 2017). Thus, a number of recent studies have highlighted the cellular processes and biological effects associated with riboflavin (Jaeger and Bosch, 2016; Schwechheimer et al., 2016; Manole et al., 2017; Naghashpour et al., 2017; Udhayabanu et al., 2017; Saedisomeolia and Ashoori, 2018). In particular, riboflavin has been shown to induce anti-nociceptive and anti-inflammatory effects in multiple experimental models of acute pain and inflammation (Franca et al., 2001; Granados-Soto et al., 2004; Bertollo et al., 2006) and has been used to treat patients with painful conditions, such as migraine (Boehnke et al., 2004), carpal tunnel syndrome (Folkers et al., 1984), diabetic polyneuropathy (Jorg et al., 1988), and premenstrual tension (Wyatt et al., 1999). In addition, one report found that the synthesis of FAD decreased in patients with amyotrophic lateral sclerosis (Lin et al., 2009). Although there are distinct subpopulations of sensory neurons and spinal circuits involved in the transduction of pain and itch stimuli, they are closely related because they are both initiated by the activation of primary sensory neurons and involve some of the same ion channels (Sun et al., 2009; Mishra and Hoon, 2013; Han and Dong, 2014). Therefore, despite the beneficial analgesic effects of riboflavin, few studies have assessed its ability to regulate anti-itch mechanisms. Thus, it would be expected that this compound would regulate the itch response. Recent reports have shown that the oral application of riboflavin reduces inflammation, thermal hyperalgesia, and formalin-induced nociception, but not tactile allodynia and motor activity based on the rotarod test in mice (Granados-Soto et al., 2004; Bertollo et al., 2006). Oral medication is the main treatment option for patients with widespread chronic itch and for those with a focal itch that is refractory to topical treatment (Mirzoyev and Davis, 2013; Maciel et al., 2014; Matsuda et al., 2016). Our results demonstrated that the oral administration of riboflavin reduced histamine-induced animal scratching (Figure 1A), although it did not change animal motor activity (Figure 1B). Further, as histamine-sensitive C-fibers cover the largest innervation area in the lower leg, the peripheral chronic itch can spread to the innervation site of damaged nerves or nerve roots (Schmelz, 2015). The primary cause of chronic pain appears to be focal neuronal discharge caused by the spontaneous or excess firing of a damaged cutaneous C-fiber after injury, scarring, or neuropathy (Sene et al., 2013). Therefore, we found that riboflavin blocked the high-frequency bursting discharge and action potentials based on histamine-sensitive single C-fiber *ex vivo* recordings (Figure 2). Riboflavin is the precursor of two active coenzyme forms in the body, FMN and FAD (Saedisomeolia and Ashoori, 2018). Riboflavin is converted to FAD through an enzymatic reaction whereby FAD synthetase adds an AMP to FMN (Schwechheimer et al., 2016; Thakur et al., 2017). These act as electron carriers in a number of redox reactions involved in energy production and in numerous metabolic pathways (Thakur et al., 2017). AMP is a well-known intermediary substance formed during the creation of energy in the form of adenosine triphosphate from food and was reported to reduce the duration of pain due to shingles and prevent the development of postherpetic neuralgia (Bednarczuk, 1986). In addition, endogenous adenosine can act as a pain suppressor, and adenosine is generated from AMP. To determine whether riboflavin or its coenzymes FMN and/or FAD have any effects on itch, we examined the effects of riboflavin, FMN, and FAD on capsaicin-induced inward currents and histamine-induced scratching *in vitro* and *in vivo*, respectively. Interestingly, we found that riboflavin and FMN attenuated histamine-induced itch behavior (Figures 1C,D, 4D) and that FMN inhibited capsaicin-induced currents in cultured mouse DRG neurons and TRPV1-transfected HEK293 cells, but that FAD did not have these effects (Figures 4, 5). This result indicates that FMN and FAD might differentially function in anti-nociception or anti-pruritus responses *via* a TRPV1-dependent pathway.

Taken together, our findings suggest that riboflavin levels, as well as FMN and FAD levels, are differentially related to histamine-induced itch and thermal-hyperalgesia responses *via* distinct mechanisms. Although many distinct compounds including chloroquine, β-alanine, serotonin, endothelin-1, and also those found in cowhage, induce itching/scratching behaviors and intracellular signals *via* distinct histamine-independent molecular pathways (Mcneil and Dong, 2012), our data demonstrated that histamine-induced scratching behaviors in mice are specifically inhibited by riboflavin. These results indicate that the anti-itching effect of riboflavin depends primarily on histamine-dependent pathways and not another pro-itch mechanism.

In summary, we have shown in this study that riboflavin has anti-pruritic effects on histamine-induced scratching behaviors, which at least in part suppresses itching behaviors. In peripheral sensory neurons, TRPV1 is further involved in the riboflavin-mediated inhibition of histamine-dependent itch. Given this, we suggest that riboflavin might serve as a novel anti-itch treatment for histamine-dependent itch.

## Data Availability Statement

The original contributions presented in the study are included in the article, further inquiries can be directed to the corresponding author/s.

## Ethics Statement

The animal study and all experimental methods were reviewed and approved by the Experimental Animal Ethics Committee of Hanyang University.

## Author Contributions

KL, YIC, and S-TI performed electrophysiological experiments and analyzed the data. H-KL and J-ZI performed behavioral tests. KL, S-MH, and YHK drafted the manuscript. SJJ and C-KP conceived and supervised the project. All authors contributed to the article and approved the submitted version.

## Conflict of Interest

The authors declare that the research was conducted in the absence of any commercial or financial relationships that could be construed as a potential conflict of interest.

## Footnotes

^1^http://dapsys.net
